# The role of clinical characteristics and pulmonary function testing in predicting risk of pneumothorax by CT-guided percutaneous core needle biopsy of the lung

**DOI:** 10.1186/s12890-021-01625-0

**Published:** 2021-08-06

**Authors:** Chunhai Li, Dexiang Wang, Fengxia Yang, Yang Song, Xuejuan Yu, Bo Liu, Haipeng Jia, Wei Zhou

**Affiliations:** 1 Department of Radiology, Qilu Hospital, Cheeloo College of Medicine, Shandong University, 107 Wenhuaxi Road, Jinan, 250012 Shandong China; 2 Department of Respiratory Medicine, Qilu Hospital, Cheeloo College of Medicine, Shandong University, 107 Wenhuaxi Road, Jinan, 250012 Shandong China; 3 Shandong Medicinal Biotechnology Center, Shandong First Medical University and Shandong Academy of Medical Sciences, 18877 Jingshi Road, Jinan, 250062 Shandong China; 4 Department of Radiation Oncology, Qilu Hospital, Cheeloo College of Medicine, Shandong University, 107 Wenhuaxi Road, Jinan, 250012 Shandong Province China

## Abstract

**Background:**

We aim to analyze the risk factors for pneumothorax associated with computed tomography (CT)-guided percutaneous core needle biopsy (PCNB) of the lung. Whether the lung function characteristics are related to pneumothorax is unclear.

**Methods:**

We retrospectively evaluated 343 patients who received CT-guided pulmonary PCNBs and underwent preoperative pulmonary function testing. Demographical, lesion-related, procedure-related features and histopathological diagnosis, as well as results of pulmonary function test were analyzed as risk factors of pneumothorax

**Results:**

Variables associated with higher rate of pneumothorax were location of lesion, presence of emphysema, and dwell time. The proportion of middle lobe, lingular, or lower lobe lesions in pneumothorax group (30/50, 60.0%) is higher than non-pneumothorax group (113/293, 38.6%). The incidence of emphysema in pneumothorax group was significantly higher than that in non-pneumothorax group (34.0% vs. 7.5%). Obstructive pulmonary function abnormalities, not restrictive, mixed ventilation function abnormalities and small airway dysfunction, correlated with pneumothorax. Multivariate logistic regression analysis showed lower location of lesion sampled and presence of emphysema were independent predictors of pneumothorax. Although dwell time, FEV_1_/FVC ratio, FEF_50%_, FEF_75%_ and FEF_25–75%_ were significantly correlated with pneumothorax on univariate analysis, these were not confirmed to be independent predictors.

**Conclusions:**

Patients with obstructive pulmonary dysfunction have a higher risk of pneumothorax. Presence of emphysema was the most important predictor of pneumothorax, followed by location of lesion.

**Supplementary Information:**

The online version contains supplementary material available at 10.1186/s12890-021-01625-0.

## Introduction

Computed tomography (CT)-guided percutaneous core needle biopsy (PCNB) of the lung has been widely considered as a common and effective procedure, and has a high degree of accuracy in clinicopathologic diagnosis. Pooled overall complication rates for PCNB and fine needle aspiration biopsy (FNAB) from 32 articles (8133 procedures) were 38.8% (95% CI 34.3–43.5%) and 24.0% (95% CI 18.2–30.8%), respectively [1]. Although overall complication rate was higher for PCNB than FNAB, there is a tendency for PCNB to replace FNAB to provide a lower false-negative rate (< 10%) in the diagnosis of pulmonary diseases [2]. As is well known, the false-negative rate of FNAB is as high as 20% in the diagnosis of lung malignant tumors. When nonspecific or inadequate tissues are biopsied, it is usually unreliable to exclude malignant diagnosis [3]. PCNB has become an important procedure to obtain enough specimens for further biological identification and molecular spectrum analysis in the individualized target therapy [4].

Pneumothorax is one of the most common complications of PCNB of the lung. Pooled pneumothorax rate for core biopsy was 25.3% (95% CI 22.2–28.6%) [1]. Previously reported common risk factors of pneumothorax for PCNBs included size, needle pleural angle, dwell time, emphysema, hyperinflation, lesion depth or intrapulmonary needle tract length, interactive breath-hold, fissure crossed, position [5–10]. The aim of this study was to investigate whether pre-procedural pulmonary function testing could help identify patients at high risk of complications. However, there is no clear clinical quantitative index of pulmonary function to estimate the risk of pneumothorax during needle biopsy. This study focused on the role of lung function in predicting the risk of pneumothorax caused by PCNB, as well as the common risk factors.

## Methods

### Patients and data collection

Three hundred and forty-three patients with pulmonary function testing have been retrospectively evaluated from 1110 consecutive patients who received CT-guided PCNBs of the lung between January 2018 and December 2019. Pulmonary function testing was performed within 7 days before PCNB. All the patients had received PCNBs for histopathological diagnosis of lung lesions in Qilu Hospital, Cheeloo College of Medicine, Shandong University, under consistent procedure. This single institutional retrospective study was approved by our hospital institutional review board (registration number: KYLL-202008-145) and complied with the Declaration of Helsinki and the ethical standards of the institutional research committee of Qilu Hospital, Cheeloo College of Medicine, Shandong University. Inclusion criteria also consisted of patients with normal electrocardiogram and with adequate hepatic, renal and hematological function. If patients were given acetylsalicylic acid, warfarin or low-molecular-weight heparin, they were required to suspend their medication 1 week before the procedure and monitor the prothrombin time. Platelet count should be ≥ 50 × 10^9^/L for biopsy.

Patient records were anonymized and de-identified prior to analysis. Collected data included patient demographics (age, gender, smoking history, prior surgery, radiotherapy or chemotherapy), characteristics of target lesions (location, the size of the sampled lesions, adjacent pleura or chest wall invasion), and procedure-related information (patient position, needle puncture site, length of biopsy pathway, dwell time, needle-pleural angle, number of needle redirections and pleural planes traversed, and number of tissue samplings), results of pulmonary function test, procedure-related complications (pneumothorax, chest drainage catheter insertion, and pulmonary hemorrhage), and the histopathological diagnosis in all cases.

**Fig. 1:**
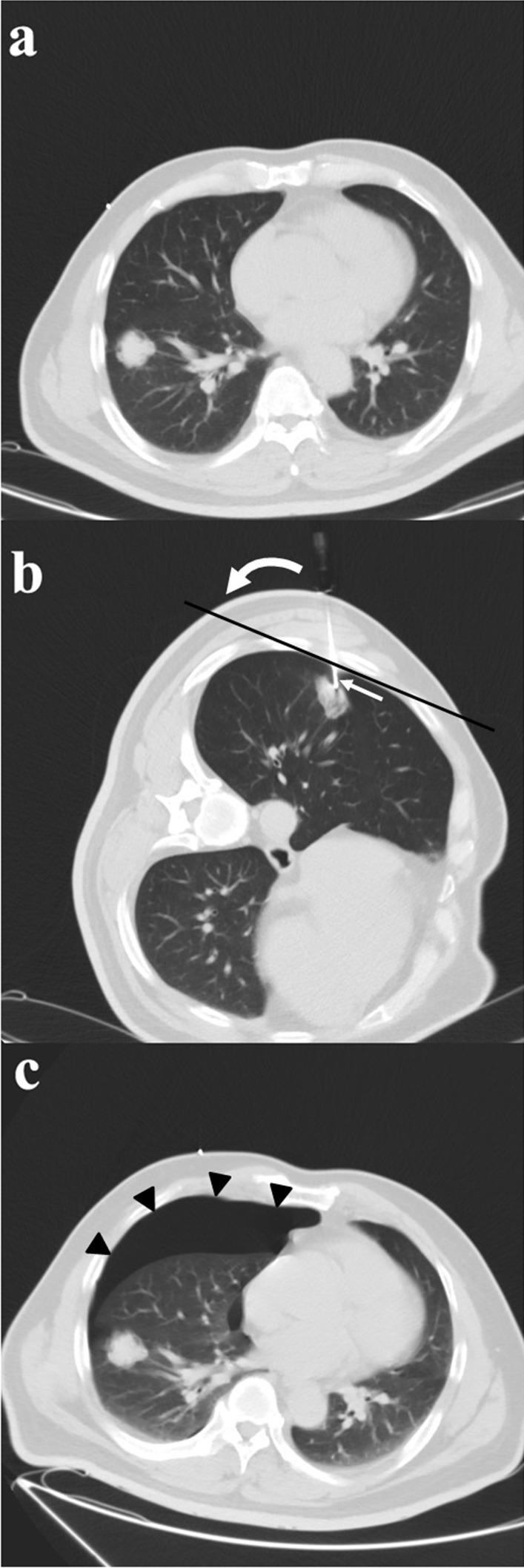
**a** CT-guided core needle biopsy of a solitary suspected lesion in the right lower lobe, in a 56-year-old male patient with 6 pack-years smoking. **b** Showed the biopsy needle (arrow) inserted within the lesion, which was later pathologically confirmed to be pulmonary adenocarcinoma. The patient was in lateral position on the CT table. The needle-pleural angle (curved white arrow), which was the minimum angle formed by a line tangent to the pleura at the puncture point and a line drawn along the needle, was 63°. The length of the needle track from the pleura to the lesion was 14.4 mm. The dwell time was 660 s. **c** CT image after the removal of the biopsy needle showed pneumothorax (arrowhead), which continued to increase until the chest drainage catheter was inserted

Middle lobe, lingular, and lower lobe lesions were categorized as “lower locations”; upper lobe lesions, as “upper locations” [11]. Lesion size was measured as the largest diameter of the sampled lesion in the previous CT images. The depth of the lesion was gauged as the length of the needle track from the punctured pleura to the edge of the lesion sampled. The needle-pleural angle was calculated on the transverse 3-mm section in the craniocaudal dimension, according to the method suggested by Ko et al*.* [6]. It was defined as the minimum angle formed by a line tangent to the pleura at the puncture point and a line drawn along the needle (Fig. 1) [6]. Pneumothorax was evaluated by CT scan after biopsy, as the largest separation between the visceral and parietal pleura. Less than or equal to 1 cm was categorized as “minor pneumothorax”; greater than 1 cm but less than or equal to 2 cm, as “intermediate pneumothorax”; greater than 2 cm, symptomatic or chest drainage catheter insertion needed, as “severe pneumothorax” [12].

### CT-guided core needle biopsy

PCNBs were performed by one intervention team led by Prof. C. L. (7 years of experience in CT-guided needle biopsy), using only one type of needle, 17-gauge coaxial introducer and 18-gauge automated cutting needle (Biopince, Argon Medical Devices, Frisco, Texas). All the biopsies were carried out according to the standard protocol. All patients underwent enhanced CT before the biopsy. Averting obvious emphysema or bulla, the safest and shortest route from the chest wall to solid part of the lesion was chosen to determine the supine, prone or lateral position of the patient on the CT table. All patients were given intravenous indwelling needles, allowing for the infusion of rescue drugs if necessary. The patients were instructed to breathe shallowly and avoid moving, coughing, speaking or deep breathing during and 3 h after the procedure. If the biopsy route needed to be changed, the patient's position could also be changed. After aseptic technique and local anesthesia with 1% lidocaine, the introducer needle was inserted. The needle is inserted rapidly during pleural puncture, and the needle is withdrawn slowly after the biopsy. Rapid insertion at breath-hold can form a precise puncture point, while slow extraction of the guide needle can make the elastic lung tissue seal the pleural hole. Then the position of the coaxial introducer was determined by CT scan. If the introducer was correctly located within the periphery of the lesion, the biopsy was performed to obtain sufficient tissue samples. The representative images of the CT-guided PCNB are shown in Fig. 1. After slowly removing the needle, CT scan was performed to evaluate the complications. The patient was then asked to rest for 24 h. Patients with pneumothorax or bleeding were monitored in the inpatient ward. Patients with intermediate or severe pneumothorax were arranged to have a follow-up CT scan to determine its stability. If patients had pneumothorax with symptoms of respiratory distress or shortness of breath, a closed thoracic drainage (8 Fr. pig-tail) was performed.

### Pulmonary function test

All patients were selected who received pulmonary function tests within 7 days before CT-guided PCNBs, in one single center of Qilu Hospital. According to the pulmonary function test, as well as their illnesses and severity, patients were mainly classified into groups of normal, small airway dysfunction, obstructive, restrictive and mixed pulmonary function abnormalities [13]. The judgment processes of pulmonary function abnormalities were referred to previous studies [13–15]. Forced expiratory volume in one second (FEV_1_)/forced vital capacity (FVC) ratio and FVC should be considered first [13, 14]. Obstructive abnormality is defined as a fixed ratio of FEV_1_/FVC < 70% [14]. Restrictive abnormality is characterized by a normal FEV_1_/FVC and a reduction in FVC [14, 15]. A mixed pulmonary function abnormality is characterized by the coexistence of obstruction and restriction.

Conventional ventilation function parameters such as FEV_1_, FEV_1_/FVC and FVC were still in the normal range, but two of these three parameters FEF_50%_, FEF_75%_ and FEF_25–75%_ were lower than 65% of the predicted value, which can be diagnosed as small airway dysfunction [15].

### Statistical analysis

After CT-guided lung biopsy, the patients were divided into two groups with or without pneumothorax. Clinical data contained characteristics associated with demographics, lesion, technique, diagnosis and pulmonary function. All quantitative data were non-normally distributed by the Shapiro–Wilk test. The Mann–Whitney U test was used for quantitative variables with non-normal distribution. The chi-square test was used to test the categorical variables. For small samples (*n* < 5), the Fisher exact test was used. Multivariate logistic regression analysis was used to identify the independent predictor of pneumothorax. Logistic regression model only contained significant covariates from univariate analysis. The variance inflation factor (VIF) was applied to measure multicollinearity. A VIF between 5 and 10 indicated high correlation that may be problematic. A two-tailed *P* value < 0.05 was defined as significant different. All statistical tests were performed by SPSS software package, standard Version 17.0 (SPSS Inc., Chicago, IL, USA).

## Results

### Comparison of groups with and without pneumothorax evaluated by univariate analysis

There were 206 (60.1%) male and 137 (39.9%) female patients in this study. Only two patients received prior chemotherapy, one received prior thoracic surgery, and no one received prior thoracic radiotherapy. The baseline characteristics of these 343 patients were summarized in Table 1. All the quantitative data showed non-normal distribution, so the median and the interquartile range (IQR) were presented here. The median age was 62 years (range 18–82 years) and the median depth from the pleura to the lesion was 11.6 mm (range 0–70.3 mm). The diameter of the lesions punctured varied from 6.8 to 212.1 mm, with a median of 26.7 mm in previous CT images. The median needle-pleural angle was measured to be 65° (range 0–90°). The median dwell time was 200 s (range 70–1200 s). Pathologically, the 248 (72.3%) malignant diagnosis mainly included 191 (55.7%) adenocarcinomas, 34 (9.9%) squamous cell carcinomas, 6 (1.7%) small cell carcinomas, and 6 (1.7%) metastases from other tumor sites (Table 2). In 80 (23.3%) benign pulmonary lesions, 71 (20.7%) were assessed as chronic pneumonia. In addition, there were 2 (0.6%) patients with borderline tumor. There were still 13 (3.8%) cases without histopathologic results for inadequate tissue sampling. Thus, primary diagnostic yield of CT-guided PCNB was 96.2%.

**Table 1:** Comparison of groups with and without pneumothorax evaluated by univariate analysis

Clinical characteristics	All patients studied		Pneumothorax			
	*N (%) or Median (lower–upper quartile)	Range^δ^	*Yes	*No	*X*^*2*^*/Z*	*P* Value^†^
No. of cases analyzed	343		50 (14.6%)	293 (85.4%)		
Demographic characteristic						
Age (years)	62 (53–68)	18–82	61 (55–65)	62 (53–68)	− 0.558	0.577
Gender					1.539	0.215
Male	206 (60.1%)		34 (9.9%)	172 (50.1%)		
Female	137 (39.9%)		16 (4.7%)	121 (35.3%)		
Pack-years smoking	0 (0–30)	0–200	6 (0–30)	0 (0–30)	− 0.752	0.452
Lesion characteristics						
Location of lesion					8.071	**0.004**
Upper	200 (58.3%)		20 (5.8%)	180 (52.5%)		
Lower	143 (41.7%)		30 (8.8%)	113 (32.9%)		
Lesion size (mm)					2.442	0.118
< 20	108 (31.49%)		11 (3.2%)	97 (28.3%)		
≥ 20	235 (68.51%)		39 (11.4%)	196 (57.1%)		
Adjacent pleura or chest wall invasion					0.057	0.812
Yes	211 (61.5%)		30 (8.8%)	181 (52.8%)		
No	132 (38.5%)		20 (5.8%)	112 (32.6%)		
Presence of emphysema					29.744	**4.929 × 10**^**–8**^
Yes	39 (11.4%)		17 (5.0%)	22 (6.4%)		
No	304 (88.6%)		33 (9.6%)	271 (79.0%)		
Technique characteristics						
Patient position					1.619	0.445
Supine	104 (30.3%)		13 (3.8%)	91 (26.5%)		
Prone	192 (56.0%)		32 (9.3%)	160 (46.7%)		
Lateral decubitus	47 (13.7%)		5 (1.5%)	42 (12.2%)		
Needle puncture site					1.559	0.816
Anterior	42 (12.2%)		7 (2.0%)	35 (10.2%)		
Anterolateral	54 (15.8%)		6 (1.7%)	48 (14.0%)		
Lateral	37 (10.8%)		5 (1.5%)	32 (9.3%)		
Posterior	165 (48.1%)		27 (7.9%)	138 (40.2%)		
Posterolateral	45 (13.1%)		5 (1.5%)	40 (11.7%)		
Length of intrapulmonary needle tract (mm)	11.6 (0.0–19.9)	0–70.3	11.0 (0.0–21.8)	11.6 (0.0–19.9)	− 0.405	0.685
Dwell time (s)	200 (169–283)	70–1200	240 (174–360)	200 (168–271)	− 1.992	**0.046**
Needle-pleural angle (°)	65 (48–80)	0–90	69 (52–85)	63 (48–80)	− 1.078	0.281
Needle redirections					1.118	0.290
Yes	31 (9.0%)		7 (2.0%)	24 (7.0%)		
No	312 (91.0%)		43 (12.5%)	269 (78.4%)		
No. of pleural punctures	1 (1–1)	1–2	1 (1–1)	1 (1–1)	− 0.492	0.622
No. of cores	2 (1–2)	1–6	2 (2–3)	2 (1–2)	− 1.014	0.311
Diagnostic characteristics					1.476	0.671
Malignant	248 (72.3%)		40 (11.7%)	208 (60.6%)		
Benign	80 (23.3%)		9 (2.6%)	71 (20.7%)		
Borderline	2 (0.6%)		0	2 (0.6%)		
Non-diagnostic/inadequate	13 (3.8%)		1 (0.3%)	12 (3.5%)		

Significant difference (*P*<0.05) are shown in bold

^*^Data are shown as number *N* (%) for categorical variables or median (lower quartile to upper quartile) for quantitative variables with non-normal distribution

^δ^Range only for quantitative variables

^†^Chi-square test for categorical variables. Mann–Whitney U test for quantitative variables. All quantitative data showed non-normal distribution by Shapiro–Wilk test

**Table 2:** Pathological types after CT guided lung puncture (343 cases)

Diagnostic characteristics	N (%)
Malignant	248 (72.3%)
Adenocarcinoma	191 (55.7%)
Squamous cell carcinoma	34 (9.9%)
Small cell carcinoma	6 (1.7%)
Metastasis from other tumor sites	6 (1.7%)
Neuroendocrine tumor	4 (1.2%)
Malignant pleural mesothelioma	3 (0.9%)
Undifferentiated carcinoma	2 (0.6%)
Adenosquamous carcinoma	1 (0.3%)
Lymphoepithelioid carcinoma	1 (0.3%)
Benign	80 (23.3%)
Chronic pneumonia	71 (20.7%)
Alveolitis	2 (0.6%)
Pulmonary hamartoma	2 (0.6%)
Tuberculosis	2 (0.6%)
Sclerosing pneumocytoma	2 (0.6%)
Neurilemmoma	1 (0.3%)
Borderline	2 (0.6%)
Solitary fibrous tumor	1 (0.3%)
Inflammatory myofibroblastic tumor	1 (0.3%)
Non-diagnostic/inadequate	13 (3.8%)

The main complications of PCNBs were pneumothorax, chest drainage catheter insertion and intrapulmonary hemorrhage. Fifty patients (14.6%) had pneumothorax after PCNBs. Among those, only three patients (6.0%) revealed severe pneumothorax on post-biopsy CT scans, which required placement of chest drainage catheter. In the most serious case of pneumothorax, the lung was compressed by 1/3. All patients with mild or moderate pneumothorax were stable without deterioration and any interventional treatment. Of all the 343 patients, 88 (25.7%) had intrapulmonary hemorrhage, most of which were slight hemorrhage.

The differences in clinical characteristics between groups with and without pneumothorax were evaluated by univariate analysis (Table 1). Variables associated with higher rate of pneumothorax were lower location (*P* = 0.004), presence of emphysema (*P* = 4.929 × 10^–8^), and dwell time (*P* = 0.046), whereas all the other parameters including demographic and diagnostic parameters showed nonsignificant findings. As shown, the proportion of lower locations in pneumothorax group (30/50, 60.0%) is higher than non-pneumothorax group (113/293, 38.6%). The dwell time varied from 70 to 1200 s, with a median of 240 s in patients with pneumothorax and 200 s in patients without pneumothorax. The incidence of emphysema in pneumothorax group was significantly higher than that in non-pneumothorax group (34.0% vs. 7.5%). However, other possible risk factors about lesion and technique, previously reported, such as lesion size, adjacent pleura, length of intrapulmonary needle tract, needle-pleural angle, and number of pleural punctures, showed no statistically significance of pneumothorax in this study.

### Differences in pulmonary function between groups with and without pneumothorax evaluated by univariate analysis

On the other hand, the pulmonary function of these 343 patients was also evaluated (Table 3). The main pulmonary function parameters were as follows: FVC(% pred) 103.3% (92.0–115.5%), FEV_1_(% pred) 98.0% (81.7–109.9%), FEV_1_/FVC ratio 76.0% (69.1–81.2%), FEF_25–75%_(% pred) 58.0% (37.4–78.6%) and peak expiratory flow (PEF% pred) 104.4% (84.4–119.6%).

**Table 3:** Differences in pulmonary function between groups with and without pneumothorax evaluated by univariate analysis*

Clinical characteristics	All patients studied		Pneumothorax			
	Median	Lower quartile –upper quartile	Yes*	No*	*Z*	*P* Value^†^
No. of cases analyzed	343		50 (14.6%)	293 (85.4%)		
Pulmonary function characteristics						
FVC (% pred)	103.3	92.0–115.5	105.0 (93.3–114.8)	103.0 (91.6–115.7)	− 0.363	0.716
FEV_1_ (L)	2.4	2.0–2.9	2.4 (1.9–2.7)	2.4 (2.0–3.0)	− 0.700	0.484
FEV_1_ (% pred)	98.0	81.7–109.9	94.8 (79.4–106.8)	98.4 (82.0–110.1)	− 1.101	0.271
FEV_1_/FVC ratio (%)	76.0	69.1–81.2	72.5 (67.1–78.4)	76.6 (69.8–81.5)	− **2.897**	**0.004**
FEF_25%_ (% pred)	88.4	64.0–106.1	73.5 (56.5–102.5)	89.5 (66.6–106.5)	− 1.950	0.051
FEF_50%_ (% pred)	72.8	48.9–94.6	58.5 (39.8–84.5)	77.5 (51.7–95.9)	− **2.458**	**0.014**
FEF_75%_ (% pred)	66.4	44.7–89.9	55.7 (42.1–79.4)	70.5 (45.4–91.1)	− **2.085**	**0.037**
FEF_25–75%_ (% pred)	58.0	37.4–78.6	44.3 (30.0–66.3)	59.6 (39.4–79.8)	− **2.134**	**0.033**
PEF (% pred)	104.4	84.4–119.6	101.5 (83.2–114.7)	104.5 (85.0–121.0)	− 1.253	0.210
MVV (% pred)	95.7	83.3–111.9	96.4 (83.6–109.2)	95.5 (83.2–112.7)	− 0.382	0.703

Significant difference (*P*<0.05) are shown in bold

^*^Data are shown as median (lower quartile to upper quartile)

^†^Mann–Whitney U test. All the quantitative data showed non-normal distribution by Shapiro–Wilk test

Furthermore, there was statistically significant difference of pulmonary function parameters between the two groups with or without pneumothorax as expressed by FEV_1_/FVC ratio (*P* = 0.004), FEF_50%_(% pred) (*P* = 0.014), FEF_75%_(% pred) (*P* = 0.037), and FEF_25–75%_(% pred) (*P* = 0.033). No difference was found in FVC between the two groups. And more importantly, as seen in Table 3, although FEV_1_ was not significantly different between the two groups of patients, FEV_1_/FVC ratio was significantly lower in patients with pneumothorax (median with IQR: 72.5, 67.1–78.4) than without pneumothorax (76.6, 69.8–81.5). Interestingly, as no other research has been studied so far, the small airway function parameters, such as FEF_50%_, FEF_75%_ and FEF_25–75%_, showed significant negative correlations with pneumothorax rate. However, maximum voluntary ventilation (MVV), PEF and FEF_25%_, which reflect the large airway function, were not associated with the risk of pneumothorax.

### Relationship between incidence of pneumothorax and pulmonary function abnormalities

Patients with pneumothorax had much lower FEV_1_/FVC ratio than those without pneumothorax, as shown above, indicating that patients with obstructive diseases were more likely to have pneumothorax. Table 4 shows the relationship between pulmonary function abnormalities and pneumothorax. The chi-square test revealed that obstructive pulmonary function abnormalities, as assessed by a decrease in FEV_1_/FVC ratio, were associated with a higher incidence of pneumothorax (*P* = 0.005). The incidence of pneumothorax in the obstructive ventilation abnormalities was 24.7%, whereas in the normal group the rate dropped to 10.7%. The restrictive and mixed ventilation function abnormalities were not found to correlate with the pneumothorax rate (*P* = 1.000 and *P* = 0.961, respectively).

**Table 4:** Relationship between incidence of pneumothorax and different types of pulmonary function abnormalities

	Pneumothorax		*X*^2^	*P* Value^†^
	Yes*	No*		
Normal ventilation function	18 (10.7%)	150 (89.3%)		
Small airway dysfunctions^δ^	10 (14.5%)	59 (85.5%)	0.670	0.413
Types of function abnormalities^δ^				
Obstructive function abnormalities	18 (24.7%)	55 (75.3%)	**7.786**	**0.005**
Restrictive function abnormalities	1 (9.1%)	10 (90.9%)	0	1.000
Mixed function abnormalities	3 (13.6%)	19 (86.4%)	0.002	0.961

Significant difference (*P*<0.05) are shown in bold

*% pred* percent predicted

^*^Data are presented as number

^δ^Each group compared with normal ventilation function group

In addition, the incidence of pneumothorax was not significantly different between patients with small airway dysfunction and patients with normal ventilation function (*P* = 0.413).

### Multivariable logistic regression model for predictors of pneumothorax in all patients studied

Multivariate logistic regression analysis was used to identify the independent predictor of pneumothorax (Table 5). Here, the analysis only considered covariables significant by univariate analysis mentioned in Tables 1 and 3. VIFs for these variables showed no multicollinearity. Lower location of lesion sampled and presence of emphysema were identified to be independent predictors of pneumothorax after CT-guided PCNB (*P* = 0.021 and *P* = 8.700 × 10^–5^, respectively). In all patients, risk of pneumothorax was significantly higher in lower location of lesion sampled (odds ratio [OR], 2.150; 95% confidence interval [CI] 1.124–4.113) and presence of emphysema (OR, 5.217; 95% CI 2.286–11.901). Although dwell time, FEV_1_/FVC ratio, FEF_50%_, FEF_75%_ and FEF_25–75%_ were significantly correlated with pneumothorax on univariate analysis, these were not confirmed to be independent risk factors here.

**Table 5:** Multivariable logistic regression model for predictors of pneumothorax in all patients studied

Multivariable Predictors*	All patients (PTX = 50, without PTX = 293)			
	VIF	Odds Ratio	95% CI	*P* value
Location of lesion	1.026	2.150	1.124–4.113	**0.021**
Presence of emphysema	1.151	5.217	2.286–11.901	**8.700 × 10**^**–5**^
Dwell time (s)	1.021	1.001	0.999–1.003	0.217
FEV_1_/FVC ratio (%)	2.133	0.995	0.938–1.056	0.865
FEF_50%_	2.574	0.984	0.995–1.015	0.307
FEF_75%_	2.168	1.012	0.987–1.037	0.349
FEF_25–75%_	1.875	1.000	0.965–1.036	0.994

Significant difference (*P*<0.05) are shown in bold

*VIF* variance inflation factor

^*^Logistic regression model only included significant covariates from univariate analysis

## Discussion

The CT-guided pulmonary PCNB can biopsy smaller pulmonary nodules with the progress of technology, but pneumothorax is still one of the most frequent complications. The rate of pneumothorax induced by PCNB was 14.6% in this study, and that of chest tube placement was 0.9%, which were similar to the rates reported in other studies [9, 16, 17]. In order to explore the risk factors of pneumothorax, clinical data such as patient demographics, characteristics of target lesions, procedure-related information, the histopathological diagnosis and results of pulmonary function test were all collected here. However, patient-related and diagnosis-related predictors had no influence on the occurrence of pneumothorax. Interestingly, Ko et al. [6] reported that pneumothorax was unlikely to occur in patients who had previous thoracic surgery, focal or diffuse pleural disease, or chest wall involvement. However, few patients in our study had prior thoracic surgery, which might contribute to the inconsistency.

Concerning lesion characteristics in this study, several predictors influenced the incidence of pneumothorax, such as the location of lesion and the presence of emphysema. Emphysema as a risk factor for pneumothorax was reported [7], but there was also different opinion [18]. Asai et al. [18] found that severity of emphysema such as stage I or II COPD, or high scores of low attenuation area (LAA) by Goddard classification were not be related to the frequency of pneumothorax. However, severe and very severe COPD patients (stage III and IV) were not included in that study. Selection bias might exist. No association was found between the incidence of pneumothorax and the depth, the size of the lesion and the invasion of adjacent pleura or chest wall, which were former risk factors for pneumothorax [5, 11, 17, 19–24]. PCNB replaced FNAB with technological advancement. For instance, several studies suggested that smaller lesion size increased the risk of pneumothorax in FNAB [17], while others found that they did not in PCNB [24]. This may be related to the increased numbers of punctures in smaller lesions with FNAB. The incidence of pneumothorax increased 2 times (66% vs. 32%) when the depth of lesion was larger than 2.0 cm, partly due to the prolongation of dwell time and the increased amount of lung tissues that the needle penetrates [17]. However, other studies on the depth of lesions and pneumothorax were quite opposite. Some studies thought that the lesion adjacent pleura or chest wall invasion was more prone to pneumothorax. Yeow et al. [24] reported that when the subpleural lesions were within 2 cm below the pleural surface, the risk of pneumothorax was sevenfold higher than the lesion depth more than 2 cm. They explained that subpleural lesions tend to shift the needle into the pleural cavity, causing air to enter [24]. We did not observe the similar phenomenon, probably because we used oblique needle approach to deal with the lesions abut pleura.

In terms of procedure-related factors, dwell time was significantly correlated with pneumothorax on univariate analysis, but it was not confirmed as an independent predictor in logistic regression model here. The longer time the needle dwelled in the lung, the more likely the respiratory movement would cause the lung to be punctured. There are both consistent and inconsistent studies [6, 7]. The dwell time span of this study was large (from 70 to 1200 s), so the effect of dwell time on pneumothorax could be better observed. Dwell time was not an independent factor, probably because it was also affected by the lung tissue structure near the needle tract and the presence or absence of emphysema. Several studies [5, 6] reported that patients with needle-pleural angles less than 80° and, in particular, less than 50°, had a higher risk of pneumothorax. However, we did not observe any impact of needle-pleural angle on complication rate of CT-guided PCNB. The negative correlation between needle-pleural angle and pneumothorax was not only due to the prolongation of intrapulmonary needle tract, but also due to the enlargement of pleural foramen torn by the needle passage [6]. The shape of the pleural aperture increased the amount of air leaking from the lung [25]. We analyzed that the reason we did not find the correlation was because we deliberately avoided oblique needle puncture in clinical practice. We have been willing to sacrifice the risk of changing the needle puncture site or prolonging the intrapulmonary needle tract in order to make the needle as perpendicular to the pleura as possible (median with IQR: 65°, 48°–80°).

Patients with obstructive pulmonary function abnormalities were at greater risk for PCNB-induced pneumothorax, which was consistent with other studies [11]. For patients with severe impairment of pulmonary function, clinicians are usually reluctant to use core needle biopsy for fear of continuous air leakage. In order to derive a quantitative index of risk for pneumothorax, pulmonary function characteristics were evaluated here. The pulmonary function index to predict PCNB-induced pneumothorax included: FEV_1_% pred, FEV_1_/FVC ratio, FVC% pred, FEF_25–75%_ (which was effort-dependent) [6, 9, 11]

However, some of these factors are inconsistent. Here, FEV_1_/FVC ratio was found to be most strongly associated with pneumothorax, which was in accordance to previous reports [6, 16, 17]. However, there were some controversies in the literature about the correlation between the abnormal pulmonary function analysis and the incidence of pneumothorax. In a retrospective analysis of 243 patients, Vitulo et al. [9] reported that no predictive value for pneumothorax was found in pulmonary function test. Several authors [6, 16–19] revealed a significant relationship between pneumothorax and FEV_1_% pred. Garcia-Rio et al*.* [16] found FEV_1_% pred, FVC% pred, FEV_1_/FVC ratio were significantly lower in patients with pneumothorax, and the closest correlation being with FEV_1_% pred. But our results were contrary to the observations of these studies. No difference was found in FEV_1_% pred between the two groups here.

Patients with obstructive functional abnormalities had significantly higher incidence of pneumothorax than those with normal or other types of pulmonary abnormalities. The increase in pneumothorax was due to poor lung elasticity, reduced alveolar air retraction, rupture of the expanded alveoli, emphysema along the needle tract and difficulty in holding breath [11, 17]. Narrowing and disappearance of small airways before the onset of emphysematous destruction can be found in COPD [26]. This might provide explanation of small airway obstruction with hyperinflation is consistent risk in many reports but emphysema is not.

Furthermore, in this study, FEF_50%_, FEF_75%_ and FEF_25–75%_ are significantly negatively correlated with the incidence of pneumothorax. Other lung function parameters such as FVC, FEF_25%_ and PEF showed no statistical difference. This study is unique because so far, no other research paper has analyzed such detailed lung function predictors for pneumothorax. FEF_50%_, FEF_75%_ and FEF_25–75%_ are the middle and end expiratory indexes of exertion dependence, which reflect small airway ventilation function. Small airway dysfunction refers to the dysfunction caused by infection, smoking and external environment in the airway with a diameter of less than 2 mm, including both small bronchi and proximal bronchioles. Small airways are known as one of the major sites of airflow obstruction in chronic obstructive pulmonary diseases (COPDs). Even before the destruction of emphysema, the narrowing and disappearance of small airways can be observed in patients with obstructive functional abnormalities, leading to the increase of peripheral airway resistance [26]. Small airway dysfunction is a common but easily ignored lung dysfunction, long known as the silent zone of the lung. Small airway lesions are characterized by smooth muscle hyperplasia and hypertrophy, inflammatory cells including neutrophils and macrophages increasing, goblet cell hyperplasia, mucus hypersecretion and obstruction, airway wall thickening and fibrosis [26]. As a result, the airflow is restricted, the alveoli are overinflated, the alveolar attachment of the small airways is destroyed, the elastic retraction of lung is reduced, and the internal pressure of the alveoli increases, thereby increasing the occurrence of pneumothorax [14, 27]. Interestingly, in this study, small airway function parameters FEF_50%_, FEF_75%_ and FEF_25–75%_, showed significant negative correlations with pneumothorax rate (Table 3). However, no significant correlation was found between small airway dysfunction and pneumothorax (Table 4). This was mainly because small airway function indicators were significantly reduced not only in small airway dysfunction group, but also in obstructive functional abnormalities group (Additional file 1: Table S1). Therefore, logistic regression analysis was further employed to evaluate the roles of pulmonary function parameters in the risk of pneumothorax, respectively (Table 5). Although FEV_1_/FVC ratio significantly correlated with pneumothorax on univariate analysis, multivariate logistic regression analysis showed no statistical significance. This indicates that FEV_1_/FVC is not an independent predictor, and may be related to other factors.

This study had several limitations. CT-guided PCNB studied here were all performed by one intervention team with only one type of needle according to the standard protocol. Some factors demonstrated no statistical significance here, which does not mean that they have nothing to do with pneumothorax. This may be due to the standardization of the procedures. Ko et al. [6] indicated that pneumothorax more commonly occurred in patients with lesions adjacent pleura or invading the chest wall. However, our puncture approach was deliberately avoided the fissure. Hence, no statistical significance of this factor was found. We used only one size of biopsy needle in this study, making it impossible to compare different sizes of the biopsy specimens. In addition, selection bias might exist. The patients studied here mainly had milder obstructive ventilatory impairment (median FEV_1_% pred 98.0% with IQR 81.7–109.9%, and median FEV_1_/FVC 76.0% with IQR 69.1–81.2%). These populations were thought to be relatively safe for PCNB and might influence the results. Furthermore, the limited reliability of this study on the risk for pneumothorax in restrictive and mixed ventilation abnormalities may be due to the limited number of such patients.

## Conclusions

Patients with obstructive pulmonary dysfunction have a higher risk of pneumothorax. Presence of emphysema was the most important predictor of pneumothorax, followed by location of lesion.

## Supplementary Information

**Additional file 1: Table S1.** Differences of small airway function parameters in obstructive function abnormalities/small airway dysfunctions group compared with normal ventilation function group.

## Data Availability

All data generated and analyzed during the current study are included in this published article.

## Abbreviations

COPD: Chronic obstructive pulmonary disease
CT: Computed tomography
FEF_25–75%_: Mean forced expiratory flow between 25 and 75% of FVC
FEF_X%_: Instantaneous forced expiratory flow when X% of the FVC has been expired
FEV_1_: Forced expiratory volume in one second
FNAB: Fine needle aspiration biopsy
FVC: Forced vital capacity
LLN: Lower limits of normal
MVV: Maximum voluntary ventilation
OR: Odds ratio
PEF: Peak expiratory flow
PCNB: Percutaneous core needle biopsy
% pred: Percent of predicted
