# Supplementary material for: The role of clinical characteristics and pulmonary function testing in predicting risk of pneumothorax by CT-guided percutaneous core needle biopsy of the lung
Source: BMC Pulm Med. 2021 Aug 6;21:257. doi: 10.1186/s12890-021-01625-0 (PMC8344205; doi:10.1186/s12890-021-01625-0)
Supplement: Supplementary file 1 — Additional file 1: Table S1. Differences of small airway function parameters in obstructive function abnormalities/small airway dysfunctions group compared with normal ventilation function group. [file 12890_2021_1625_MOESM1_ESM.docx]

**Additional file 1: Table S1 Differences of small airway function parameters in obstructive function abnormalities/small airway dysfunctions group compared with normal ventilation function group**

|  | ***N* (%)** * | **FEF_50%_ (% pred)** | | | **FEF_75%_ (% pred)** | | | **FEF_25–75%_ (% pred)** | | |
| --- | --- | --- | --- | --- | --- | --- | --- | --- | --- | --- |
|  |  | **Median**^⸹^ | ***Z*** | ***P*** | **Median**^⸹^ | ***Z*** | ***P*** | **Median**^⸹^ | ***Z*** | ***P*** |
| **Normal ventilation function** | 168 (49.0%) | 94.8 (84.3~111.9) |  |  | 89.50 (77.0~105.5) |  |  | 79.0 (65.7~92.0) |  |  |
| **Obstructive function abnormalities**^†^ | 73 (21.3%) | 39.8 (26.7~54.6) | -12.218 | 2.505×10^-34^ | 39.6 (29.0~53.6) | -11.409 | 3.760×10^-30^ | 30.0 (21.6~39.1) | -12.216 | 2.567×10^-34^ |
| **Small airway dysfunctions**^†^ | 69 (20.1%) | 62.9 (53.0~71.8) | -10.889 | 1.305×10^-27^ | 52.2 (46.1~59.0) | -10.653 | 1.687×10^-26^ | 50.3 (43.2~57.4) | -10.910 | 1.037×10^-27^ |

* Data are displayed as the number *N* (%), as the percentage of all patients studied.

^⸹^ Data are shown as median (lower quartile to upper quartile) for quantitative variables with non-normal distribution.

^†^ Each group compared with normal ventilation function group. Mann-Whitney U test.
